# Editorial: Physiological education: preparing for the future

**DOI:** 10.3389/fphys.2024.1380314

**Published:** 2024-02-15

**Authors:** J. García-Estañ, J. M. A. Oliveira, L. M. Rodrigues

**Affiliations:** ^1^ Depto. Fisiología, Fac. Medicina, Centro de Estudios en Educación Médica, Universidad de Murcia, Murcia, Spain; ^2^ UCIBIO - Applied Molecular Biosciences Unit, Faculty of Pharmacy, University of Porto, Porto, Portugal; ^3^ CBIOS–Universidade Lusofona’s Res Center Biosciences and Health Technologies, Lisboa, Portugal

**Keywords:** physiology, education determinants, teaching and learning strategies, methodologies, assessment

The Future of Physiology, frequently centered in education, has been a recurrent theme in various *physiology fora*. From profession definition to innovation and knowledge creation, it is currently recognized as essential. By this Research Topic Frontiers accepts its actuality and offers an opportunity to update and exchange experiences and views, to share new approaches and strategies, facilitating communication between stakeholders, students included. Education will continue to be key to strengthen this community gathered by this passion for physiology, more and more essential in human health education and research.

The first article discusses the Physiology future in North America from an European view This P-MIG focus group, gathering various experienced physiologists lead by the authors, identified several key determinants that might shape the coming years (Rodrigues et al.). An interesting exercise, already replicated in Europe, revealing similar concerns and perspectives within these communities.

The second article addresses learning and assessment strategies for a better humanized medical education (Tutor et al.). Exploring specific and transversal competencies for physicians, using a rubric-based assessment, the authors propose such competencies to help raise students’ awareness of the development of a more humanized medicine, allowing better responses to patients’ needs.

In the third article, Kuang discusses the concept of homeostasis in physiology. Every professor has used homeostasis to start the teaching of physiology and talk about important processes such as temperature and blood glucose regulation, pH balance, and the control of blood pressure. The mechanisms involve stabilizing and adapting regulation coordinated by a hierarchical web of control loops. The effect/consequence includes the purpose of protectiveness and the side effect of hiding some health issues. The article introduces the term “homeostatic tendency” to unite the stability and adaptability of homeostasis, which is significant because it unites both terms, either toward stabilizing the previous steady state or achieving a new one through adaptation. The article concludes with insights into why students struggle with learning homeostasis.

In the fourth article, Carrasco-Gómez et al. present an interesting study with gamification to improve the teaching of physiology, using an escape room activity. The study included a qualitative survey to assess the students’ perceptions of the activity, their preferences for peer-to-peer teaching and traditional methods, and their feedback on the escape room experience. Additionally, the study analysed the participants’ performance in a final exam to compare the effectiveness of the two teaching methods. 98% of the students found the activity positive and advantageous for their learning. The majority preferred peer-to-peer teaching over traditional methods and the participants group had a higher academic performance in the final exam compared to the non-participants group. Gamification works!

In the fifth article, Heber et al. report a successful intervention to improve conceptual knowledge of medical students who rely on memorization of disclosed items. The project aimed to steer student learning away from the memorization of isolated facts toward the acquisition of conceptual knowledge by defining 45 learning objectives in physiology. The results showed improved learning outcomes, specifically at the competence level compared to Research Topic that were not included. The effect nearly doubled the probability for a correct answer regarding physiological concepts. Student feedback indicates a high level of agreement that this project significantly improved their understanding of physiological concepts, and supported their learning for the summative exam. The study also highlights the importance of integrating knowledge and acquiring conceptual understanding in medical education.

In the sixth article, dealing with active methodologies, Cardozo et al. showed that active learning methodology and formative assessment effectively reduced stress and anxiety while improving knowledge, in this case of cardiac physiology. These authors combined active learning strategies to provide feedback and help students identify areas requiring improvement. The effectiveness of the methodology was compared to the traditional method in reducing stress and anxiety by measuring cortisol and α-amylase, and the STAI test (for anxiety). The results showed that the active methodology, with formative assessment, decreased test stress and anxiety, and improved student performance compared to traditional lectures. However, the study could not demonstrate an effect of the methods used to improve long term learning and retention.

In the seventh article, the authors describe the assessment of a workshop attended by secondary education students and undergraduate students of medicine and nursing degrees (García-Durán et al.). The workshop was supervised and accompanied by the instructors, and it took place at the laboratory facilities of the University. The assessment by undergraduate students showed that the overall rating of the workshop stations was quite good and rated the organization and the material used in each station very positively. Regarding the skills or competencies, they improved thanks to the participation in the workshop; 38% of the students mentioned improved oral communication skills, followed by self-esteem and interpersonal relationship. The students also expressed a high level of agreement regarding the positive impact of the workshop on their academic background, and they believed that similar activities should be offered in other courses of the degree. No significant differences were observed between male and female students in their responses.

Finally, a nice paper using flipped classroom (FC) methodology, Ganfornina et al. investigated the potential benefits of applying FC in a Neurophysiology module. The authors compared standard lecturing with FC, where they used a printed student Workbook as pre-class material for each session. Their approach differed from standard FC by relying on student’s internal motivation and by performing no pre-class/in class assessment. The authors report that the FC groups showed a significant increase in average scores. Also, by using a sentiment analysis of student perceptions assessed with open-ended surveys, the authors conclude that students appreciate the FC strategy, being particularly positive for the understanding of physiology concepts.

